# Medically assisted reproduction and the risk of being born small and very small for gestational age: Assessing prematurity status as an effect modifier

**DOI:** 10.3389/fphar.2022.904885

**Published:** 2022-09-28

**Authors:** Jessica Gorgui, Odile Sheehy, Jacquetta Trasler, Anick Bérard

**Affiliations:** ^1^ Research Center, CHU Sainte-Justine, Montreal, QC, Canada; ^2^ Faculty of Pharmacy, University of Montreal, Montreal, QC, Canada; ^3^ Departments of Pediatrics, Human Genetics and Pharmacology and Therapeutics, McGill University, Montreal, QC, Canada; ^4^ Research Institute of the McGill University Health Centre, McGill University, Montreal, QC, Canada

**Keywords:** assisted reproduction, perinatal health, perinatal adverse outcomes, obstetrics-high risk, pharmacoepidemiology, effect modifier, epidemiology methods, prematurity

## Abstract

Over the last decade, the use of medically assisted reproduction (MAR) has steadily increased but controversy remains with regards to its risks. We aimed to quantify the risk of being born small for gestational age (SGA) and very SGA (VSGA) associated with MARs overall and by type, namely ovarian stimulators (OS) and assisted reproductive technology (ART). We conducted a cohort study within the Quebec Pregnancy Cohort. Pregnancies coinciding with Quebec’s MAR reimbursement PROGRAM period (2010–2014) with a singleton liveborn were considered. MAR was first defined dichotomously, using spontaneous conception as the reference, and categorized into three subgroups: OS alone (categorized as clomiphene and non-clomiphene OS), ART, OS/ART combined. SGA was defined as being born with a birth weight below the 10th percentile based on sex and gestational age (GA), estimated using populational curves in Canada, while VSGA was defined as being born with a birth weight below the 3rd percentile. We then estimated odds ratios (OR) for the association between MAR and SGA as well as VSGA using generalized estimated equation (GEE) models, adjusted for potential confounders (aOR). Two independent models were conducted considering MAR exposure overall, and MAR subgroup categories, using spontaneous conceptions as the reference. The impact of prematurity status (less than 37 weeks gestation) as an effect modifier in these associations was assessed by evaluating them among term and preterm pregnancies separately. A total of 57,631 pregnancies met inclusion criteria and were considered. During the study period, 2,062 women were exposed to MARs: 420 to OS alone, 557 to ART, and 1,085 to OS/ART combined. While no association was observed between MAR and SGA nor VSGA in the study population, MAR was associated with an increased risk for SGA (aOR 1.69, 95% CI 1.08–2.66; 25 exposed cases) among preterm pregnancies; no increased risk of SGA was observed in term pregnancies. MARs are known to increase the risk of preterm birth and our results further confirm that they also increase the risk of SGA among preterm pregnancies.

## Introduction

Infertility affects 11.5%–15.7% of women (Bushnik et al., 2012); 8%–20% of couples reported having difficulties conceiving (Hull et al., 1985; Thonneau et al., 1991; Case, 2003; Oakley et al., 2008), and 8%–30% of infertility remain unexplained (European Society for Human Reproduction and Embryology, 1996). Fertility treatments are defined as procedures of medically assisted reproduction (MAR) and include *in vitro* fertilization (IVF), intrauterine insemination (IUI), and ovarian stimulators (OS) (Zegers-Hochschild et al., 2009). We refer to procedures handling oocytes and/or sperm, or embryos to induce a pregnancy as assisted reproductive technology (ART) (Zegers-Hochschild et al., 2009).

In August of 2010, Quebec was the first Canadian province to put in place a universal reimbursement program for MAR. Through the implementation of this program, decision makers aimed to 1) help infertile/subfertile couples procreate, 2) reduce multiplicity with the application of a single embryo transfer policy, and 3) increase Quebec’s birth rate (Salois, 2014). The program was halted in October of 2014 following a higher than expected rise in healthcare expenditure. While the program was active in Quebec, no surveillance program was put in place, and as such this made it difficult to establish patterns of MAR use and the impact on these methods on both maternal and perinatal health. Although the Canadian ART Register (CARTR) is in place, it mainly focuses on ART which would exclude OS and IUI, which are both widely used as first line practice to induce pregnancy.

More than eight million children have been conceived specifically through IVF worldwide since the first IVF baby, Louise Brown, was conceived in 1978 (reported in 2019) (Fauser, 2019). A systematic review looking at existing registries reporting ART utilization has described the trends between 2004 and 2013 (Kushnir et al., 2017). During this period, across centers including Australia, the United Kingdom, the United States, Canada, and Japan, over seven million ART cycles resulting in over 1.4 million live-births have been reported (Kushnir et al., 2017). More specifically, CARTR reports demonstrates that ART use has steadily increased, having more than tripled in the last decade (Gunby, 2011), reporting 35,347 cycles in 2019 and 30,764 in 2020 across Canada (CFAS, 2020). ART-conceptions have significantly increased because of the universal reimbursement program; specifically, 2% of all Quebec pregnancies resulted from IVF in 2012-13 versus 1.2% in 2009-10 (Salois, 2014). It was thus foreseeable that all MARs have increased during this time-period in Quebec.

In 2016, 8% of children were born small for their gestational age (SGA) in Canada (Shiraz El Adam et al., 2022). SGA is a composite measure of gestational age and birth weight. A child born SGA ranks among the lowest 10th percentile for their gestational-age specific birth weight according to population-based references (Kramer et al., 2001). Using the same references, a child born VSGA ranks among the lowest 3rd percentile (Kramer et al., 2001). Given that SGA and VSGA are composite measures that account for gestational age at birth, they are known to be a better marker for child development during pregnancy, as opposed to measuring birth weight alone, for example, (Ananth and Platt, 2004).

Our team established that MARs increase the risk of prematurity when compared to spontaneous conception, which is also a known association in the literature (Gorgui and Bérard, 2018; Gorgui et al., 2020). However, the association between MARs and SGA is not well studied and there is limited information on non-IVF MARs such as OS alone and ART methods. For example, when comparing IVF-conceived singletons to those who were spontaneously conceived, studies observed a 1.4-1.6 fold increase in the risk of SGA among IVF singletons (Helmerhorst et al., 2004; Jackson et al., 2004; Katalinic et al., 2004). An additional study published in the United Kingdom found that IVF significantly increased the risk of SGA by two-fold when compared to spontaneous conception (Governent of the United Kingtom, 2010).

MAR conceptions remain on the rise and given the changes in the political landscape in Quebec and the possibility of a new reimbursement program is on the political agenda. Given the significant consequences of SGA and VSGA on children’s health and development, our aim was to quantify the association between MARs and SGA primarily as well as VSGA. Additionally, given the known association between prematurity status and MAR (Gorgui et al., 2020), we aimed to assess if prematurity was an effect modifier in these associations. Our hypothesis is that MARs may be associated with an increased risk of SGA and/or VSGA specifically among those born preterm. Lastly, we aimed to quantify this association specifically among women exposed to OS, ART, and OS/ART combined to adjust for confounding by indication, namely infertility/subfertility (Malloy, 2002).

## Materials and methods

### Data source

We conducted a cohort study within the Quebec Pregnancy Cohort (QPC). The QPC is a population-based cohort with prospective data collection which is built through the linkage of three Quebec databases; namely, 1) Régie de l’Assurance Maladie du Québec (RAMQ), which includes medical services/procedures and pharmaceutical service database (including drug name, start date, dosage, duration, prescribers), 2) MED-ECHO, which includes hospitalization archives data [International Classification of Disease—9th/10th revision (ICD-9 and 10) diagnostic codes, interventions, procedures, and consultations, gestational age], and lastly 3) Institut de la Statistique du Québec (ISQ), which includes sociodemographic data, birth weight, and gestational age. Through a unique patient encrypted identifier, data from each of these databases were linked. All pregnancies of women covered by the Quebec public prescription drug insurance plan that have occurred between 01/1998 and 12/2015 are included in the QPC. Data on mothers and children following the end of pregnancy are also collected, as such, the QPC provides a prospective follow-up from at least 1 year prior to the first day of gestation (1DG), during the entirety of the pregnancy, and until 12/2015. The 1DG is defined as the first day of the last menstrual period. This information is validated against ultrasound measures, which are obtained through patients’ charts (Vilain et al., 2008). The QPC and its’ data sources are described in further detail in Berard and Sheehy (2014).

### Study population

A pregnancy was eligible if the date of conception occurred between 05/08/2010 and 15/11/2014; was covered by the RAMQ drug plan 1 year before and during pregnancy; and resulted in a singleton liveborn. We specifically chose to study the time-period of 08/2010-11/2014, as the Quebec universal MAR reimbursement program was active at that time. Multiple pregnancies were excluded, because MARs increase the risk of multiplicity and as such could be a potential effect modifier in the association between MAR and SGA, as it is in the causal pathway of the association between MAR and prematurity (Goldenberg et al., 2008). In addition, given that single embryo transfer was enforced during the Quebec MAR reimbursement period, we aimed to study the association between MAR and SGA within a real life experiment. We excluded pregnancies exposed to known fetotoxic medications during pregnancy (Supplementary Table S1) (Koren et al., 1998; Kulaga et al., 2009).

### Study design

A cohort study was performed within eligible pregnancies in the QPC.

### Exposure

MAR was defined as any procedures including egg harvesting, IVF, IUI or at least one prescription filling for OS (clomiphene, estradiol, progesterone, gonadotropins, chorionic gonadotrophin, leuprolide, citorelix, ganirelix, follitropin, choriogonadotropin-α) occurring within 2 months prior to and 1 month after the 1DG (Supplementary Tables S2, S3). We chose to include a 2-month time-window prior to the 1DG to ensure that the studied pregnancy resulted from the identified MAR procedure or OS use. Additionally, we added 1 month following the 1DG to account for late billings by physicians, as the QPC contains data collected mainly for reimbursement purposes (RAMQ for prescription filling and MAR procedures).

We first assessed MAR overall and then categorized MAR in three subgroups as OS alone, ART alone, and OS/ART combined, using pregnancies with spontaneous conception as the reference. Subsequently, we also stratified OS use alone as clomiphene only users and non-users, which is the most commonly prescribed OS in the clinical setting.

### Outcome

SGA is a composite measure of birth weight and gestational age. We identified cases of SGA by using data on gestational age at delivery as well as birth weight and newborn sex. Gestational age and birth weight have been validated against patients’ charts (Vilain et al., 2008). SGA was defined as newborns being among the lowest 10th percentile for birth weight according to gestational age and sex using Canadian population-based references (Kramer et al., 2001). Additionally, we looked at very SGA (VSGA) which is defined as newborns being among the lowest 3rd percentile of birth weight according to gestational age and sex using Canadian population-based references (Kramer et al., 2001). Prematurity status was defined using the definition by the World Health Organisation based on gestational age at birth which had to occur before 37 completed weeks of gestation (World Health Organization, 2016). We used the MedEcho database validated against measures in patients charts in addition to the statistics database in order to define SGA (Vilain et al., 2008).

### Covariates

We selected the following potential covariates based on their association with the use of MAR or because they have been reported as risk factors for SGA: 1) Sociodemographic variables on the 1DG including maternal age, receipt of welfare, area of residence (urban vs. rural); 2) Previous pregnancy in the year before the 1DG, ending in delivery, abortion or miscarriage; 3) Maternal history of chronic comorbidities during the year before the 1DG and until the end of the 1st trimester, namely hypertension and diabetes as diagnoses for these conditions are often only obtained at the start of a clinical follow-up (i.e., during pregnancy follow-up with a general practitioner or obstetrician), 4) Depression/anxiety, asthma, thyroid disorders, epilepsy, coagulopathies, infections and other medication use for conditions other than those described were measured in the year before the 1DG; 5) Obesity and smoking were measured during the year before the 1DG and during pregnancy as these variables are likely reported at prenatal visits. We used a combination of ICD-9 and ICD-10 codes as well as prescription fillings related to the studied health conditions to measure all covariates pertaining to maternal conditions described above in section (4) (Supplementary Table S4).

### Pregnancy complications

Premature birth and therefore SGA/VSGA may also occur due to a number of complications in pregnancy such as premature rupture of membranes, placental dysfunction or preterm labor. MAR-pregnancies are at an increased risk for these complications (Nagata et al., 2019), and as such these variables are in the causal pathway of the studied association. Though we are unable to adjust for them in our multivariate models, we have measured them in compared groups in order to assess if they may be involved in the obtained results.

### Statistical analyses

We performed descriptive statistics to compare MAR conceived and spontaneous conception pregnancies in terms of covariate status. The unit of analysis was a pregnancy. We performed *t*-tests and X^2^ for continuous and categorical variables, respectively. Pregnancy complications [premature rupture of membranes, placental dysfunction, preterm labor (Supplementary Table S5)] were compared between groups. We estimated crude as well as adjusted odds ratios (ORs and aORs) and 95% confidence intervals (95% CI) to measure the association between MAR and SGA as well as VSGA, with spontaneous conception as reference, using generalized estimated equation (GEE) models. Adjustments were performed to account for potential confounding variables identified above. Using GEE models allows us to account for inter- and intra-pregnancy variability as women could have contributed more than one pregnancy during the study period. Furthermore, in order to assess if prematurity status is an effect modifier in these associations, we performed our main analyses in a cohort comprised of term births and in a cohort comprised of preterm births. By definition, effect modification would occur if the association between MAR and SGA as well as VSGA differs depending on third variable, which in this instance would be prematurity status (Rothman et al., 2008). We hypothesized that babies born preterm would be more at risk of being born SGA and/or VSGA.

In our secondary analysis, we estimated the association between SGA and categories of MAR, namely OS alone (subsequently categorized as clomiphene users vs. non-users), ART alone, ART/OS combined and the risk of SGA. We also estimated the association between subcategories of MAR (OS alone, ART alone, ART/OS combined) and VSGA. For this secondary analysis, we used spontaneous conception as the reference. This sub-categorized analysis was the first method we used to account for the underlying subfertility/infertility, as the severity would differ among exposure categories.

Lastly, we performed sensitivity analyses within a sub-cohort of MAR-exposed women to account for potential confounding by the underlying subfertility/infertility, which would be the main indication for conception through MARs. The restriction to this sub-cohort allows to determine if the association between MAR and SGA is independent of subfertility/infertility. Statistical analyses were performed using SAS (SAS Institute Inc., Version 9.4, Cary, NC).

### Ethics approval

This study was approved by the Quebec Data Access Agency (Commission d’accès à l’information—CAI) and the CHU Sainte-Justine Institutional Review Board. Additionally, the CAI has authorized the linkage between databases composing the QPC.

## Results

**FIGURE 1:**
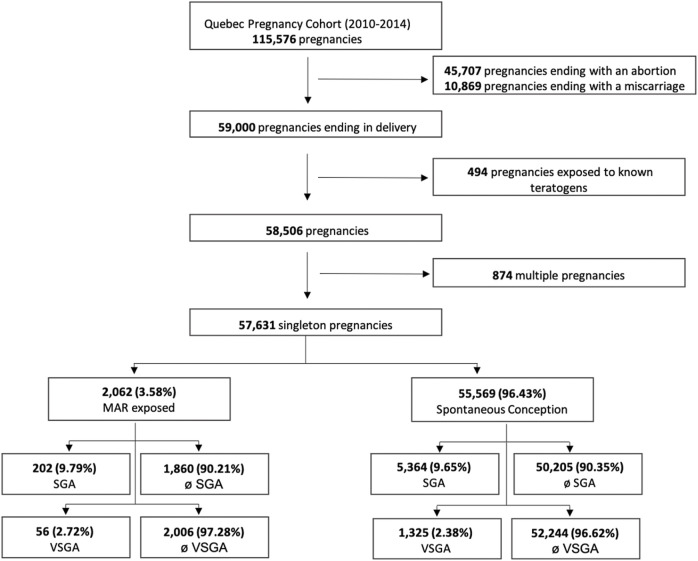
Flowchart of the selection process of the study population. MAR, medically assited reproduction; SGA, small for gestational age; VSGAI, very small for gestational age.

**FIGURE 2:**
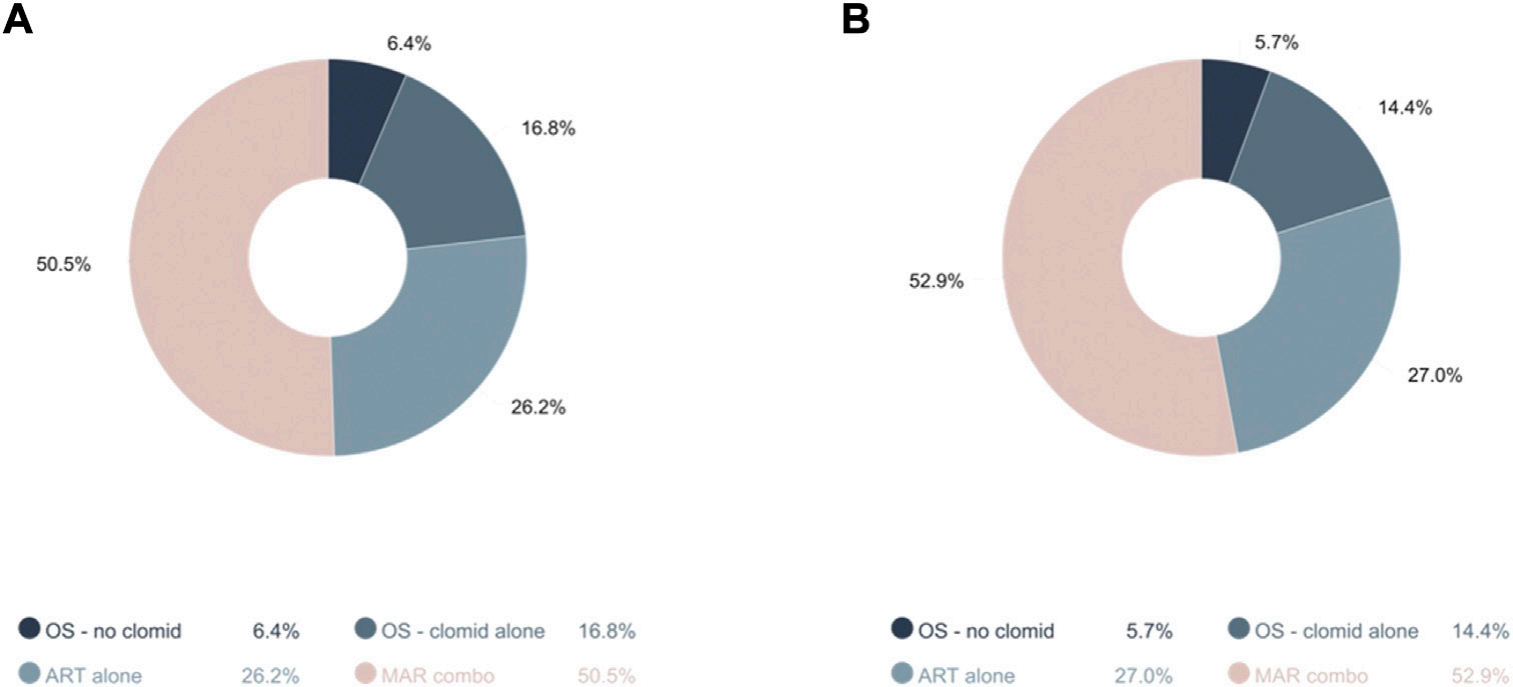
Distribution of MAR categories among **(A)** pregnancies resulting in SGA (*n* = 202) and **(B)** non-SGA babies (*n* = 1,860). Legend: ART, assisted reproduction techniques; MAR, medically assisted reproduction; OS, ovarian stimulators; SGA, small for gestational age.

Overall, 57,631 singleton pregnancies met inclusion criteria and were considered for analyses; 2,062 (3.6%) were pregnancies conceived through MARs and 55,569 (96.4%) through spontaneous conception (Figure 1). Among all MAR conceptions, 420 (20.4%) women were exposed to OS alone among which 302 women were exposed to clomiphene only, 557 (27.0%) to ART, and 1,085 (52.7%) to OS/ART combined (Figure 2). Among OS alone users, the majority used clomiphene [302 (71.90%)]. Among MAR conceptions, 202 (9.81%) resulted in SGA babies while 5,364 (9.65%) resulted in SGA among those who did not have MAR (Figure 1).

Among MAR conceptions, women were more likely to be older (≥35 years old), welfare recipients, which are known risk factors for prematurity, low birth weight and as such SGA (Table 1) than women with spontaneous conception (SC). MAR conceived babies were born more preterm and with lower birth weights (Table 1). No differences were observed across profiles of maternal comorbidities (e.g., depression, anxiety, epilepsy) which are known risk factors for SGA, except for the history of polycystic ovarian syndrome (Table 1). There were no differences between MAR conceptions and SC women in regard to complications during the current pregnancy (premature rupture of membranes, placental dysfunction, and PT labor—Table 1) nor in their patterns of utilization of healthcare services, which we measured through the use of medication (any medication that was not used to define a comorbidity as above) as well as the follow-up by obstetrician or hospitalization/emergency visit (Table 1). Of note, given that variables measured during pregnancy are more likely to be in the causal pathway between MAR and SGA such as pregnancy complications (e.g., premature rupture of membranes, placental dysfunction, PT labor) (Nagata et al., 2019) we did not adjust for them in all subsequent models.

**TABLE 1:** Characteristics of the study population.

	MAR conception	Spontaneous conception	*p*-value^a^
	(n = 2,062)	(n = 55,569)	
Sociodemographic characteristics			
Maternal characteristics			
Maternal age, years—(mean ± SD)	32.64 ± 5.37	29.01 ± 5.60	**<0.001**
Maternal age, years			
<25	4 (0.19)	441 (0.79)	
25–35	147 (7.13)	12,369 (22.26)	
35–40	1,111 (53.87)	33,015 (59.41)	
≥40	800 (38.80)	9,744 (17.54)	**<0.001**
Welfare recipient	227 (11.01)	10,621 (19.11)	**<0.001**
Urban dweller	1,775 (86.21)	45,908 (82.61)	**<0.001**
Pregnancy and child characteristics			
Pregnancy characteristics			
Preterm delivery (<37 weeks gestation)	183 (8.87)	3,496 (6.29)	**<0.001**
Low birth weight (<2500 g)	141 (6.84)	2,787 (5.02)	**<0.001**
Small for gestational age (<10th percentile)	202 (9.81)	5,364 (9.65)	0.81
Very small for gestational age (<3rd percentile)	56 (4.06)	1,325 (2.38)	**<0.001**
Gestational age at delivery (weeks)	38.65 ± 2.05	38.86 ± 1.73	**<0.001**
Child characteristics			
Male sex	1,063 (51.63)	28,559 (51.39)	0.83
Birth weight, grams—(mean ± SD)	3,298.43 ± 583.70	3,340.26 ± 526.63	**<0.001**
Maternal comorbidities measured in the 12 months before the 1DG^b^			
Diabetes	72 (3.50)	2,187 (3.94)	0.31
Hypertension	25 (1.21)	681 (1.23)	0.96
Obesity	42 (2.04)	1332 (2.40)	0.30
Asthma	164 (7.97)	4,965 (8.93)	0.13
Epilepsy	25 (1.21)	647 (1.16)	0.84
Smoking dependence	38 (1.85)	1,098 (1.98)	0.67
Infection	629 (30.55)	16,296 (29.32)	0.23
Polycystic ovarian syndrome	6 (0.29)	62 (0.11)	**0.02**
Thyroid disease	77 (3.74)	2,421 (4.36)	0.18
Depression/anxiety	247 (12.00)	7,180 (12.92)	0.22
Coagulopathy	12 (0.58)	211 (0.38)	0.25
Previous pregnancy	227 (11.02)	6,631 (11.93)	0.21
Any other medication use^c^			
None	1,446 (70.13)	39,661 (71.37)	
1	386 (18.72)	9,814 (17.66)	
2-3	189 (9.17)	4,934 (8.88)	
4+	41 (1.98)	1,160 (2.09)	0.48
Pregnancy complications measured in the 12 months before the 1DG			
Premature rupture of membranes	103 (5.00)	3,114 (5.60)	0.24
Placental dysfunction	13 (0.63)	293 (0.53)	0.52
Preterm Labor	21 (1.02)	551 (0.99)	0.90
Bleeding	43 (2.09)	1,353 (2.43)	0.32
Utilization of healthcare services			
Follow-up by obstetrician,^d^	1,210 (58.77)	31,984 (57.55)	0.27
Follow-up by general practitioner or specialist^d^	1,223 (59.40)	31,925 (57.45)	0.08
Hospitalization and/or emergency visit^e^	818 (39.73)	21,311 (39.73)	0.21

Legend: 1DG, first day of gestation; ART, assisted reproduction techniques; MAR, medically assisted reproduction; OR, odds ratio; OS, ovarian stimulators.

Bold values represent significant, *p* < 0.005.

a *p* value calculated to compared term births to preterm births using Pearson χ2 test for categorical variable and a *t* test for continuous variables.

b Diagnoses are based on ICD-10 codes and/or a filled prescription in relation to the comorbidity.

c Excludes all prescription fillings included in the definitions of all considered comorbidities above.

d Defined as five visits or more during the course of the pregnancy.

e During the 12 months before the 1DG.

### Association between medically assisted reproductions and small for gestational age in the main cohort

Adjusting for potential confounders, we found no association between MAR conception and the risk of SGA (aOR 1.08, 95% CI 0.93–1.25, 202 exposed cases) when compared to spontaneous conception (Table 2). We additionally included a propensity score prediction model using the same variables as those used in the multivariate model and found the same result (aOR 1.09, 95% CI 0.94–1.27, 202 exposed cases) (Table 2). Upon categorizing the exposure to MARs, we found that the exposure to OS alone seemed to have a stronger association, although not statistically significant (aOR 1.23, 95% CI 0.91–1.66, 47 exposed cases) (Table 2). Furthermore, when recategorizing OS exposure as clomiphene use or other OS, we can see that the association between clomiphene, the most used OS, and SGA is the strongest when compared to SC, without reaching the desired level of statistical significance (aOR 1.22, 95% CI 0.86–1.73, 34 exposed cases) (Table 3).

**TABLE 2:** Use of medically assisted reproduction and the risk of being born small for gestational age overall and by subtype among the main cohort (*n* = 57,631).

	SGA (*n* = 5,565)	No SGA (*n* = 52,065)	Crude OR (95% CI)	Adjusted OR* (95% CI)	Adjusted OR** (95% CI)
MAR use overall					
Spontaneous conception	5,364 (96.39)	50,205 (96.43)	1.00	1.00	1.00
MAR conception	202 (3.61)	1,860 (3.57)	1.01 (0.87–1.17)	1.08 (0.93–1.25)	1.09 (0.94–1.27)
MAR use by subtype					
Spontaneous conception	5,364 (96.39)	50,205 (96.43)	1.00	1.00	—
ART alone	53 (0.95)	504 (0.96)	1.00 (0.76–1.32)	1.10 (0.83–1.45)	
OS alone	47 (0.84)	373 (0.72)	1.20 (0.89–1.62)	1.23 (0.91–1.66)	
OS and ART combined	102 (1.83)	983 (1.89)	0.94 (0.76–1.16)	1.01 (0.82–1.24)	

Legend: 1DG, first day of gestation; ART, assisted reproduction techniques; CI, confidence interval; MAR, medically assistedreproduction; OR, odds ratio; OS, ovarian stimulators. *Adjusted for sociodemographic variables (maternal age, urban dwelling, welfare recipient) as well as maternal comorbidities measured within 12 months prior to the 1DG (hypertension, diabetes, polycystic ovarian syndrome, asthma, epilepsy, depression/anxiety, coagulopathy, infection, and other medication use) and during pregnancy (smoking, obesity). **Adjusted including propensity score prediction using the same variables as those used in the multivariate model.

**TABLE 3:** Use of medically assisted reproduction and the risk of being born small for gestational age based on a secondary classification of exposure among the main cohort (*n* = 57,632).

MAR use by subtype	SGA (*n* = 5,566)	Crude OR (95% CI)	Adjusted OR* (95% CI)
Spontaneous conception	5,364 (96.37)	1.00	1.00
OS alone (excluding clomid)	13 (0.23)	1.17 (0.67–2.06)	1.16 (0.66–2.05)
Clomid alone	34 (0.61)	1.21 (0.85–1.72)	1.22 (0.86–1.73)
ART alone	53 (0.95)	1.00 (0.76–1.32)	1.05 (0.79–1.42)
OS and ART combined	102 (1.83)	0.94 (0.76–1.16)	0.96 (0.78–1.18)

Legend: 1DG, first day of gestation; ART, assisted reproduction techniques; CI, confidence interval; MAR, medically assisted reproduction; OR, odds ratio; OS, ovarian stimulators; SGA, small for gestational age. *Adjusted for sociodemographic variables (urban dwelling, welfare recipient) as well as maternal comorbidities measured within 12 months prior to the 1DG (polycystic ovarian syndrome) and during the 1st trimester of pregnancy (hypertension, diabetes).

### Association between medically assisted reproductions and very small for gestational age in the main cohort

Adjusting for potential confounders, we found no association between MAR conception and the risk of VSGA (aOR 1.20, 95% CI 0.92–1.58, 56 exposed cases) when compared to SC (Table 4). Upon categorizing the exposure to MARs, we found that the exposure to OS alone significantly increased the risk of VSGA (aOR 1.66, 95% CI 1.01–2.72, 16 exposed cases) (Table 5).

**TABLE 4:** Use of medically assisted reproduction and the risk of being born very small for gestational age overall and by subtype among the main cohort (*n* = 57,631).

MAR use overall	VSGA (*n* = 1,381)	No VSGA (*n* = 56,251)	Crude OR (95% CI)	Adjusted OR* (95% CI)
Spontaneous conception	1,325 (95.94%)	54,244 (96.43%)	1.00	1.00
MAR conception	56 (4.06%)	2,006 (3.57%)	1.14 (0.87–1.50)	1.20 (0.92–1.58)

Legend: 1DG, first day of gestation; ART, assisted reproduction techniques; CI, confidence interval; MAR, medically assistedreproduction; OR, odds ratio; OS, ovarian stimulators. *Adjusted for sociodemographic variables (maternal age, urban dwelling, welfare recipient) as well as maternal comorbidities measured within 12 months prior to the 1DG (hypertension, diabetes, asthma, depression/anxiety, infection, and other medication use) and during pregnancy (obesity).

**TABLE 5:** Use of medically assisted reproduction and the risk of being born very small for gestational age based on a secondary classification of exposure among the main cohort (*n* = 57,632).

MAR use by subtype	VSGA (*n* = 1,381)	No VSGA (*n* = 1,381)	Crude OR (95% CI)	Adjusted OR* (95% CI)
Spontaneous conception	1,325 (95.94%)	52,244 (96.43%)	1.00	1.00
OS	16 (1.16%)	407 (0.72%)	1.62 (0.99–2.66)	1.66 (1.01–2.72)
ART alone	3 (0.22%)	147 (0.26%)	0.85 (0.27–2.64)	0.92 (0.29–2.87)
OS and ART combined	37 (2.68%)	1,452 (2.58%)	1.03 (0.74–1.43)	1.10 (0.79–1.54)

Legend: 1DG, first day of gestation; ART, assisted reproduction techniques; CI, confidence interval; MAR, medically assisted reproduction; OR, odds ratio; OS, ovarian stimulators; VSGA, very small for gestational age. *Adjusted for sociodemographic variables (urban dwelling, welfare recipient) as well as maternal comorbidities measured within 12 months prior to the 1DG (asthma, epilepsy, thyroid disease, depression/anxiety, and other medication use) and during the 1st trimester of pregnancy (hypertension, diabetes).

### Prematurity status as an effect modifier in the association between medically assisted reproduction and small for gestational age as well as very small for gestational age

To assess if prematurity status is an effect modifier in the studied associations, we performed our analyses stratified on this status. No association was identified between MAR and SGA (aOR 1.03, 95% CI 0.88–1.21, 177 exposed cases) (Table 6a) nor between MAR and VSGA (aOR 1.15, 95% CI 0.89–1.62, 48 exposed cases) in the term cohort (Table 7a). However, in the preterm cohort, MAR was associated with a significantly increased risk of SGA (aOR 1.69, 95% CI 1.08–2.66, 25 exposed cases) (Table 6b). Though we did not observe a significant association between MAR and VSGA in the preterm cohort likely due to lack of power, we do observe results in the same range (aOR 1.61, 95% CI 0.76–3.42, eight exposed cases) (Table 7b). These results suggest that prematurity status is indeed an effect modifier in the association between MAR and SGA as well as VSGA given the difference in the point estimates compared to those obtained in the main unstratified cohort (results above).

**TABLE 6:** Use of medically assisted reproduction and the risk of being born small for gestational age stratified among term(a) and preterm (b) births (*n* = 57,631).

(a) Term cohort (*n* = 53,952)	SGA (*n* = 5,200)	No SGA (*n* = 48,752)	Crude OR (95% CI)	Adjusted OR* (95% CI)
MAR use overall				
Spontaneous conception	5,023 (96.6%)	47,050 (96.5%)	1.00	1.00
MAR conception	177 (3.4%)	1,702 (3.5%)	0.97 (0.83–1.14)	1.03 (0.88–1.21)
**(b) Preterm cohort** (*n* = 3,679)	**SGA (*n* = 366)**	**No SGA (*n* = 3,313)**	**Crude OR (95% CI)**	Adjusted OR* (95% CI)
MAR use overall				
Spontaneous conception	341 (93.2%)	3,155 (95.2%)	1.00	1.00
MAR conception	25 (6.8%)	158 (4.8%)	1.46 (0.95–2.58)	1.69 (1.08–2.66)

Legend: 1DG, first day of gestation; ART, assisted reproduction techniques; CI, confidence interval; MAR, medically assistedreproduction; OR, odds ratio.*Adjusted for sociodemographic variables (maternal age, urban dwelling, welfare recipient) as well as maternal comorbidities measured within 12 months prior to the 1DG (hypertension, diabetes, asthma, depression/anxiety, infection, and other medication use) and during pregnancy (smoking, obesity).

**TABLE 7:** Use of medically assisted reproduction and the risk of being born very small for gestational age stratified among term (a) and preterm (b) births (*n* = 57,631).

(a) Term cohort (*n* = 53,952)	VSGA (*n* = 1,267)	No VSGA (n = 52,685)	Crude OR (95% CI)	Adjusted OR** (95% CI)
MAR use overall				
Spontaneous conception	1,219 (96.21%)	50,845 (96.52%)	1.00	1.00
MAR conception	48 (3.79%)	1,831 (3.48%)	1.09 (0.81–1.45)	1.15 (0.89–1.62)
**(b) Preterm cohort (*n* = 3,679)**	**VSGA (*n* = 114)**	**No VSGA (n = 3,565)**	**Crude OR (95% CI)**	Adjusted OR** (95% CI)
MAR use overall				
Spontaneous conception	106 (92.98%)	3,390 (95.09%)	1.00	1.00
MAR conception	8 (7.02%)	175 (4.91%)	1.46 (0.70–3.05)	1.61 (0.76–3.42)

Legend: 1DG, first day of gestation; CI, confidence interval; MAR, medically assisted reproduction; OR, odds ratio; VSGA, very small for gestational age. **Adjusted for sociodemographic variables (urban dwelling, welfare recipient) and maternal age.

### Confounding by indication

To address confounding by indication in the main association, we performed two sensitivity analyses in which we performed the same main analyses as shown above in a restricted study cohort of women exposed to MARs overall (*n* = 2,062). Similarly to the results reported above, we found no association between exposure to any subcategory of MAR and the risk of SGA (Tables 8, 9).

**TABLE 8:** Use of medically assisted reproduction and the risk of being born small for gestational age by subtype among a cohort of women exposed to medically assisted reproduction (*n* = 2,062).

MAR use by subtype	SGA (*n* = 202)	Crude OR (95% CI)	Adjusted OR* (95% CI)
OS alone	47 (23.27)	1.00	1.00
ART alone	53 (26.24)	0.84 (0.55–1.27)	0.85 (0.56–1.29)
OS and ART combined	102 (50.50)	0.83 (0.57–1.19)	0.83 (0.58–1.20)

Legend: 1DG, first day of gestation; ART, assisted reproduction techniques; CI, confidence interval; MAR, medically assistedreproduction; OR, odds ratio; OS, ovarian stimulators; SGA, small for gestational age. *Adjusted for sociodemographic variables (urban dwelling, welfare recipient) as well as maternal comorbidities measured within 12 months prior to the 1DG and during the 1st trimester of pregnancy (hypertension and diabetes).

**TABLE 9:** Use of medically assisted reproduction and the risk of being born small for gestational age based on a secondary classification of exposure among a cohort of women exposed to medically assisted reproduction (*n* = 2,062).

MAR use by subtype	SGA (*n* = 202)	Crude OR (95% CI)	Adjusted OR* (95% CI)
OS alone (excluding clomid)	13 (6.44)	1.00	1.00
Clomid alone	34 (16.83)	1.02 (0.52–2.01)	1.04 (0.52–2.08)
ART alone	53 (26.24)	0.85 (0.45–1.62)	0.88 (0.46–1.68)
OS and ART combined	102 (50.49)	0.84 (0.46–1.54)	0.86 (0.47–1.59)

Legend: 1DG, first day of gestation; ART, assisted reproduction techniques; CI, confidence interval; MAR, medically assisted reproduction; OR, odds ratio; OS, ovarian stimulators; SGA, small for gestational age. *Adjusted for sociodemographic variables (urban dwelling, welfare recipient) as well as maternal comorbidities measured within 12 months prior to the 1DG and during the 1st trimester of pregnancy (hypertension and diabetes).

## Discussion

The prevalence of SGA in our study cohort was (5,566/57,631) 9.66% 9.80% overall, which is higher than the last reported prevalence of 6.10% in Canada. This finding could be attributed to both increased prematurity and MAR conceptions during the study period, specifically in our study population (CARTR Canadian, 2016; Gorgui et al., 2020).

Adjusting for potential confounders, we found no significant association between MAR and SGA as well as VSGA in the main cohort (Tables 2, 4). According to a systematic review and meta-analysis conducted by Jackson *et al.* SGA increased by 1.6-fold (OR 1.6, 95% CI 1.3–2.0) with IVF-conceptions compared to SC (Helmerhorst et al., 2004; Jackson et al., 2004). It is important to note however that some individual studies had strong significant associations, while a number of other studies found no association similarly to our current findings (Helmerhorst et al., 2004; Jackson et al., 2004). As such, our findings are in line with the literature and further add to the body of evidence to support an association between MAR and VSGA, which has not yet been studied. We performed sensitivity analyses in order to adjust for potential confounding by the underlying infertility. Results were the same as in our main analyses, suggesting that our findings are robust (Tables 7, 9). Additionally, we performed a *post hoc* sample size calculation and determined that we have more than the needed sample (*n* = 984) to observe a significant difference of 2% between groups. For reference, the incidence of SGA in IVF (included in our MAR subgroup) pregnancies is estimated around 8%, while it is estimated around 4% in the spontaneously conceived pregnancies (Slavov et al., 2021), hence the conservative choice for a 2% difference in the power calculation above.

When looking at the main cohort, we did not find an association between categories of MARs and SGA, though OS seem to be playing a role in an increased risk of SGA (aOR 1.23, 95% CI 0.91–1.66, 47 exposed cases) (Table 2). Through our analyses, we saw that OS use increases the risk of VSGA (aOR 1.66, 95% CI 1.01–2.72, 16 exposed cases) (Table 5) and may also be playing a role in the association between MARs and SGA (aOR 1.23, 95% CI 0.91–1.66, 47 exposed cases) (Table 2) Exposure to OS has been associated with SGA when compared with SC (RR, 1.71; 95% CI: 1.09–2.69) (Governent of the United Kingtom, 2010), as well as both with (Chung et al., 2006; Mitwally et al., 2006; Imudia et al., 2012) and without IVF (van der Spuy et al., 1988; D’Angelo et al., 2011) yielding similar results. It has been hypothesized in this context that alteration in oocyte quality, decreased receptivity of the endometrium or the production of a poor implantation environment may play a role in this finding. These could be mediated in part through increased estradiol levels, which would impair the implantation process (Kondapalli and Perales-Puchalt, 2013). This hypothesis has been further been confirmed in animal studies (Kondapalli and Perales-Puchalt, 2013).

Basing ourselves on the fact that we had previously identified an association between MAR and prematurity in our data (Gorgui et al., 2020) and knowing that prematurity and SGA/VSGA have the same risk factors, we acknowledge the fact that prematurity may be an effect modifier in the association between MAR and SGA/VSGA. This was imperative to assess as both outcomes increase morbidity and mortality in children. To our knowledge, this study is the first to assess the impact of the prematurity in this association. In fact, our results have demonstrated that the prematurity status is indeed an effect modifier in the association between MAR and SGA as well as VSGA (Tables 6, 7). This is a novel finding in the context of the era of MAR use and suggests that it may be clinically important to make the distinction between MAR babies born term and preterm when assessing their perinatal outcomes, including SGA/VSGA. To further support our conclusion, a study conducted by Clausson et al. (1998) using the Swedish Medical Birth Register first identified the importance to subdivide SGA status based on gestational age as they observed higher mortality rates among preterm-SGA babies.

### Strengths and limitations

Through the QPC, the outcomes and exposures we measure have previously been validated. MARs were defined as a prescription filling or medical procedures. Our research team has previously validated prescription fillings for antidepressants and antibiotics among others against maternal reports in the QPC (positive and negative predictive values > 87%) (Zhao et al., 2017). Though we are aware that prescription fillings do not exactly reflect treatment intake and that we have not specifically validated OS use, we believe that in the context of infertility where the desire to get pregnant is present, we are measuring our exposure to OS appropriately. Furthermore, we used procedure codes to defined MARs (excluding OS) which are reliable given that they are used for billing purposes by physicians. Additionally, gestational age, which defines our main outcomes in part, has been validated (Vilain et al., 2008). We have also used the most updated population-based reference in Canada for growth curves to measure SGA/VSGA (Kramer et al., 2001) and used the birth weight which is obtained through the ISQ. This data has been compared to medical records and found to be reliable (Vilain et al., 2008; Berard and Sheehy, 2014).

Though we have adjusted for a number of potential confounders, it is important to understand that due to the nature of the analysis, some relevant variables of parameters occurring during pregnancy cannot be taken into account. In the context of the studied association, variables such as infections, premature rupture of membranes, placental issues could be relevant to account for, as they may explain slower development *in utero* and consequently affect birth weight, but are in the causal pathway between MARs and SGA. However, in order to measure the potential impact of these variables, we compared them between our exposure groups and did not find any differences (Table 1). As such, we believe that accounting for these variables is unlikely to modify our estimates.

Our study is limited by the absence of information on the underlying causes of infertility and on the paternal implications in the couple’s infertility as this is a mother-child cohort. Additionally, it is difficult to diagnose infertility and for the most part is poorly reported, especially when considering hat 30% of cases remain unexplained (European Society for Human Reproduction and Embryology, 1996). Despite the lack of information on the reasons for infertility due to the nature of the collected data, we aimed to address the potential for indication bias this by performing a number of sensitivity analyses among a sub-cohort of women exposed to MARs, and found similar results to those obtained in the main cohort. This suggests that despite accounting for the underlying infertility through this cohort restriction, no association exists between MARs and SGA prior to stratification on prematurity status.

The universal reimbursement program for MAR allowed an important number of women insured by the public program for their medications (usually of lower socioeconomic status) to resort to MARs. We are aware that the generalizability of our results could be affected as the QPC is not able to capture MAR exposures in the private sector. The private sector grants access to MARs to those with higher family incomes and therefore more likely to have private insurance for their medication. As such, the QPC is unable to capture these women and their exposure. Though this would allow for a higher sample size, we believe that the impact of this on the generalizability of our results would be minimal as our team has demonstrated that women insured by the public and private sectors had similar profiles, through a validation study (Berard and Lacasse, 2009).

## Conclusion

Conception through MAR was not associated with an increased risk of SGA nor VSGA compared to SC in the main cohort. However, prematurity status was revealed to be an effect modifier in this association as MAR increased the risk of SGA among preterm birth. Given the continuous rise in infertility and MAR use as well as the changes in the current political landscape which could lead to increased access to these methods, it is important for physicians and their patients to be aware of the particularity of babies born preterm, which additionally may lead them to have an increased risk of being born SGA.

## Data Availability

The original contributions presented in the study are included in the article/Supplementary Material, further inquiries can be directed to the corresponding author.
