# Supplementary material for: Medically assisted reproduction and the risk of being born small and very small for gestational age: Assessing prematurity status as an effect modifier
Source: Front Pharmacol. 2022 Sep 28;13:904885. doi: 10.3389/fphar.2022.904885 (PMC9554408; doi:10.3389/fphar.2022.904885)
Supplement: Supplementary file 1 [file DataSheet1.docx]

**Supplementary data**

This supplementary data has been provided by the authors to give readers additional information about their work.

Supplementary data to:

 **Medically Assisted Reproduction and the Risk of Being Born Small and Very Small for Gestational Age: Assessing Prematurity Status as an Effect Modifier**
Jessica Gorgui, Odile Sheehy, Jacquetta Trasler, Anick Bérard.

List of Supplemental Tables

[Table S1. List of known fetotoxic medications. 3](#_Toc536803856)

[Table S2. Definition of medically assisted reproduction exposure based on procedure codes. 4](#_Toc536803857)

[Table S3. Definition of ovarian stimulator exposure based on Quebec generic medication codes. 5](#_Toc536803858)

[Table S4. List of diagnostic codes (ICD-9 and ICD-10) and medications used for the identification of maternal comorbidities 6](#_Toc536803859)

[Table S5. List of diagnostic codes (ICD-9 and ICD-10) for pregnancy complication compared in Table 1. 14](#_Toc536803860)

[Table S6. Simulation of post-hoc power calculations^1^ for the sensitivity analysis taking into account confounding by indication. 15](#_Toc536803861)

# Table S1. List of known fetotoxic medications.

| Class | Common Denomination |
| --- | --- |
| Systemic retinoids | Isotretinoin, acitretin, etretinate, tretinoin (retinoic acid), retinol, retinal, adapalene, bexarotene, tazarotene |
| Antiepileptics | Acetazolamide, carbamazepin, eslicarbazepine, ethosuximide, gabapentin, lacosamide, lamotrigine, levetiracetam, oxcarbazepine, perampanel, phenobarbital, phenytoin, pregabalin, primidone, valproic acid, rufinamide, sodium divalproex, sodium valproate, topiramate, sodium valproate, stiripentol, tiagabine, topiramate, vigabatrin, zonisamide |
| Anti-thyroid drugs | Methimazole, propylthiouracil |
| Anti-coagulants | Warfarin |
| Tetracycline derivative | Nicoumalone, doxycyclin, minocyclin, streptomycyn, tetracycline |
| Aangiotensin converting enzyme (ace) inhibitors | Benazepril, captopril enalapril, fosinopril, lisinopril, perindopril, quinalapril, ramipril, trandolapril, verapamil |
| Androgens | Danazol, testosterone, methyltestosterone |
| Antineoplastic agents | Anastrozole, bicalutamide, busereline, busulfan, chlorambucil, cyclophosphamide, estramustine, etoposide, exemestane, fluorouracile, flutamide, gosereline, hydrosyuree, ifosfamide, interferon alfa-2B, interferon alfa-2B, letrozole, melphalan, mercaptopurine, methotrexate, nilutamide, procarbazine, tamoxifen, thioguanine, tretinoin, triptoreline |
| Others | Amiodarone, diethylstilbestrol, dihydroergotamine, fluconazole, leflunomide, lithium (carbonate de), lithium (citrate de), methotrexate, penicillamine, cyclophosphamide, misoprostol, diclofenac/misoprostol, quinine |
| Statins | Atorvastatin, amlodipine/atorvastatin, cerivastatin, fluvastatin, lovastatine, lovastatin/nicotinic acid, pravastatin, pravastatin /ASA, rosuvastatin, simvastatin |
| Angiotensin-receptor blocker (arb) | Candersartan, candersartan/HCTZ, eprosartan, eprosartan/HCTZ, irbesartan, irbesartan/HCTZ, losartan, losartan/HCTZ, olmesartan, olmesartan/HCTZ, telmisartan, telmisartan/HCTZ, valsartan, valsartan/HCTZ |

# Table S2. Definition of medically assisted reproduction exposure based on procedure codes.

| Procedure code | Definition |
| --- | --- |
| 06954-57 | Sperm bank use |
| 06962-64 | IVF with Ovarian stimulation |
| 06965-67 | IVF with Egg harvesting |
| 06968 | Fresh embryo transfer |
| 06969 | Frozen embryo transfer |
| 06972 | Ovarian stimulators – no IVF |
| 06975 | Ovarian stimulators – no IVF |
| 06976 | Egg Harvesting |
| 06971, 06973 | Artificial insemination – no IVF |
| 06993-95 | Artificial insemination |

Legend: IVF – *in vitro* fertilization

# Table S3. Definition of ovarian stimulator exposure based on Quebec generic medication codes.

| Medication | Quebec Generic Code |
| --- | --- |
| Leuprolide | 4632 46632 46632 |
| Citorelix | 47813 |
| Ganirelix | 47818 |
| Follitropine | 45612  47815  47508  47810 |
| Choriogonadotropine | 4316 |
| Chloridrate gonadoreline | 40901 |
| Gonadoreline | 47837 |
| Gonadotrophins | 47809 |
| Chlorio alpha | 47811 |
| Progesterone | 8151 46362 46276 |
| Estradiol | 34232 45488  46417  46489  46766 |
| Clomifene | 47863  2080  47814 |

# Table S4. List of diagnostic codes (ICD-9 and ICD-10) and medications used for the identification of maternal comorbidities

|  | |
| --- | --- |
| Hypertension | |
| Diagnostic codes:  ICD-9 codes: 401.0-405.9, 642.0-642.9 and 796.2  ICD-10 codes: I100, I101, I150, I151, I152, I158, I159, O10, O11, O12, O13, O14, O15 and O16 | |
| Medication generic codes:  Generic name | Quebec generic code |
| Clonidine | 10751 |
| Methyldopa | 6136 |
| Hydralazine | 4524 |
| Minoxidil | 41564 |
| Doxazosine | 45625 |
| Prazosin | 37742 |
| Terazosin | 45520 |
| Acebutolol | 45463 |
| Atenolol | 43670 - 46325 - 46315 |
| Bisoprolol | 47355 |
| Carvedilol | 47199 - 46319 |
| Labetalol | 45243 |
| Metoprolol | 38275 - 46763 - 46780 |
| Nadolol | 40563 |
| Oxprenolol | 42162 |
| Pindolol | 39016 |
| Pindolol-HCTZ | 45408 |
| Propranolol | 8229 |
| Sotalol | 44866 |
| Timolol | 38314 |
| Amlodipine | 47006 |
| Felodipine | 45624 |
| Nifedipine | 42708 - 46388 - 46469 |
| Nifedipine-AAS | 47751 |
| Nimodipine | 45532 |
| Diltiazem | 43228 - 47247 |
| Cilazapril | 47056 |
| Cilazapril-HCTZ | 47320 |
| Spironolactone | 9100 - 46572 |
| Ethacrynique | 3562 |
| Furosemide | 4173 |
| Amiloride | 41759 |
| Amiloride-HCTZ | 41772 |
| Hydrochlorothiazide | 4537 |
| Chlorthalidone | 1976 |
| Indapamide | 43397 |
| Metolazone | 19440 |
| Amiloride-HCTZ | 41772 |
| Spironolactone-HCTZ | 38158 |
| Triamterene-HCTZ | 38197 |
| Triamterene | 9763 |
| Aliskirene | 47706 |
| Aliskirene-HCTZ | 47823 |
| Diabetes | |
| Diagnostic codes:  ICD-9 codes: 250.0-250.9, 271.4 and 790.2  ICD-10 codes: E10-E14 and R73 |  |
| Medication generic codes:  Generic name | Quebec generic code |
| Metformin | 5824 - 47208 |
| Glucagon | 4238 |
| Chlorpropamide | 1937 |
| Glyburid | 4264 |
| Tolbutamide | 9672 - 15184 |
| Gliclazide | 46056 - 47329 |
| Glimepiride | 46799 - 47427 |
| Acarbose | 46300 - 47151 |
| Pioglitazone | 46678 - 47392 |
| Rosiglitazone | 47371 - 46642 |
| Rosiglitazone/Metformin | 46862 |
| Rosiglitazone/Glimepiride | 47652 |
| Nateglinide | 46810 |
| Repaglinide | 47357 - 46568 |
| Saxagliptine | 47817 |
| Sitagliptine | 47715 |
| Sitagliptine/Metformin | 47807 - 47832 |
| Insulin aspart | 46798 - 47424 |
| Insulin aspart/ Insulin aspart protamine |  |
| Insulin glulisine | 47749 |
| Insulin isophane bio-synthetic | 44164 |
| Insulin lispro | 46322 - 47206 |
| Insulin crystal zinc bio-synthetic | 44489 |
| Insulin crystal zinc and isophane bio-synthetic | 45531 |
| Insulin aspart/insulin aspart protamine | 47615 |
| Insulin detemir | 47586 |
| Insulin glargine | 47536 |
| Insulin lispro/insulin lispro protamine | 47426 |
| Insulin globine zinc | 4823 |
| Insulin sulfate | 4888 |
| Insulin crystal zinc (porc) | 18296 |
| Insulin protamine zinc (beef) | 18309 |
| Insulin protamine zinc (porc) | 18322 |
| Insulin isophane (porc) | 18335 |
| Insulin isophane (beef) | 18348 |
| Insulin slow release (beef and porc) | 39120 |
| Insulin isophane (beef and porc) | 39133 |
| Insulin protamine zinc (beef and porc) | 39146 |
| Insulin semi-slow release (beef and porc) | 39159 |
| Insulin ultra-slow release (beef and porc) | 39172 |
| Insulin crystal zinc (beef and porc) | 39185 |
| Insulin isophane (beef)* | 39458 |
| Insulin protamine zinc (beef)* | 39484 |
| Insulin protamine zinc (porc)* | 39497 |
| Insulin crystal zinc (beef)* | 39523 |
| Insulin slow release (porc) | 41655 |
| Insulin crystal zinc (porc)/ insulin isophane (porc) | 43033 |
| Insulin crystal zinc (beef) | 43735 |
| Insulin isophane semi-biosynthetic of human sequence | 44151 |
| Insulin slow release semi-biosynthetic of human sequence | 44476 |
| Insulin crystal zinc semi-biosynthetic of human sequence | 44502 |
| Insulin ultraslow release semi-biosynthetic of human sequence | 44996 |
| Insulins isophane and crystal zinc semi-biosynthetic of human sequence | 45405 |
| Insulin slow release biosynthetic of human sequence | 45415 |
| Insulin ultraslow release biosynthetic of human sequence | 45483 |
| Insulins isophane and crystal zinc biosynthetic of human sequence | 45511 |
| Insulins crystal zinc and isophane semi-biosynthetic of human sequence | 45534 |
| Insulin crystal zinc (beef and porc) | 46536 |
| Insulin isophane (beef and porc) | 46537 |
| Insulin slow release (beef and porc) | 46538 |
| Insulin isophane(human)/ insulin injectable(human) | 46592 |
| Insulin isophane (human) | 46602 |
| Insulin injectable (human) | 46603 |
| Insulin lispro/insulin isophane (human) | 46607 |
| Insulin crystal zinc (porc)* | 47004 |
| Insulin lispro/ insulin lispro protamine | 47426 |
| Asthma | |
| Diagnostic codes:  ICD-9 codes: 493.0, 493.1, 493.3-493.9  ICD-10 codes: E10-E14 and R73 | |
| Medication generic codes:  Generic name | Quebec generic code |
| Aminophylline | 364, 46428 |
| Beclomethasone | 780 |
| Budesonide | 45499 |
| Budesonide/ formoterol | 47428, 46800 |
| Sodium Chromoglycate | 39419, 47315 |
| Disodium cromoglycate | 2223 |
| Epinephrine | 3380 |
| Epinephrine | 3406 |
| Epinephrine racemic | 3419 |
| Fenoterol | 38548 |
| Flunisolide | 38730 |
| Fluticasone | 47050, 46435 |
| Formoterol | 47231 |
| Formoterol | 47271, 46430 |
| Formoterol / budesonide | 47428 |
| Ipratropium (bromide) | 43124, 46640 |
| Ipratropium (bromide)/ salbutamol (sulfate) | 47186, 46302 |
| Isoproterenol (chlorhydrate) | 5083 |
| Isoproterenol (chlorhydrate)/ phenylephrine (bitartrate) | 5096 |
| Isoproterenol (chlorhydrate)/ phenylephrine (chlorhydrate) | 5109 |
| Isoproterenol (sulfate) | 5070 |
| Ketotifen (fumarate) | 45555, 46752 |
| Sodium Montelukast | 47303, 47302, 46467 |
| Sodium Nedocromil | 47033, 45563, 46463 |
| Orciprenaline (sulfate) | 6721 |
| Oxtriphylline | 43475 |
| Pirbuterol (acetate) | 47153, 46299 |
| Procaterol hemihydrate (chlorhydrate) | 45547 |
| Salbutamol | 10530 |
| Salbutamol (sulfate) | 33634, 46737 |
| Salmeterol (xinafoate)/fluticasone (propionate) | 47335, 46597 |
| Salmeterol (xinafoate) | 47112, 46247 |
| Terbutaline (sulfate) | 34180 |
| Theophylline | 9464, 46847, 9490, |
| Theophylline/dextrose | 44944 |
| Triamcinolone (acetonide) | 9737 |
| Zafirlukast | 47266, 46401 |
| Ciclesonide | 47626 |
| Momethason | 45581 |
| Momethason/Formeterol |  |
| Zolair |  |
| Depression and anxiety | |
| Diagnostic codes:  ICD-9 codes: 296, 309, 311, 300.0 and 300.4  ICD-10 codes: F30, F31, F32, F33, F34, F38, F39, F40, F41 and F43 |  |
| Medication generic codes:  Generic name | Quebec generic code |
| Citalopram | 46543 - 47317 |
| Escitalopram | 47553 |
| Fluoxetine | 45504 |
| Fluvoxamine | 45633 |
| Paroxetine | 46164 - 47061 |
| Sertraline | 45630 |
| Duloxetine | 47714 |
| Venlafaxine | 46244 - 47118 |
| Isocarboxazid | 5018 |
| Phenelzine | 7280 |
| Tranylcypromine | 9698 |
| Amitriptyline | 442 - 429 - 46836 |
| Amoxapine | 43696 |
| Clomipramine | 14781 |
| Desipramine | 2522 |
| Doxepin | 3198 |
| Imipramine | 4784 |
| Maprotiline | 37443 |
| Nortriptyline | 46835 - 6578 |
| Protriptyline | 8294 |
| Trimipramine | 9906 |
| Bupropion | 46435 - 47285 |
| Buspirone | 45609 |
| Maprotiline | 37443 |
| Mirtazapine | 46744 - 47408 |
| Trazodone | 43137 |
| Moclobemide | 46427 - 47005 |
| L-tryptophane | 42058 |
| Nefazodone | 46235 - 47093 |
| Thyroid diseases | |
| Diagnostic codes:  ICD-9 codes: 244.0-244.9 and 242.9  ICD-10 codes: E01, E02, E03 and E05 |  |
| Medication generic codes:  Generic name | Quebec generic code |
| Levothyroxine (sodium) | 5252 - 46574 |
| Liothyronine (sodium)/levothyroxine (sodium) | 33842 |
| Liothyronine (sodium) | 5317 - 46457 - 46474 |
| Methimazole | 40836 |
| Propylthiouracil | 8242 |
| Epilepsy | |
| Diagnostic codes:  ICD-9 codes: 345.0-345.9  ICD-10 codes: G400-G419 |  |
| Medication generic codes:  Generic name | Quebec generic code |
| Carbamazepine | 1404 |
| Ethosuximide | 3757 |
| Divalproex | 44073 |
| Gabapentin | 46229, 47100 |
| Lamotrigin | 46248, 47110 |
| Levetiracetam | 47463 |
| Oxcarbazepin | 46805, 47430 |
| Pregabalin | 47571 |
| Valproate | 39393 |
| Topiramate | 46359, 47229 |
| Valproic acid | 38951 |
| Vigabatrin | 46477, 47080, 46211 |
| Phenobarbital | 17906, 19128, 46558 |
| Phenytoin | 43449, 43462 |
| Idelalisib | 8047 |
| Coagulopathy | |
| Diagnostic codes:  ICD-9 codes: 286.0-286.9, 641.3, and 286  ICD-10 codes: D66, D67 |  |
| Medication generic codes:  Generic name | Quebec generic code |
| Fibrinogen | 40342 |
| Human Plasma – coagulatory factors | 40368 |
| Human Plasma – coagulatory factors | 41811 |
| Human Plasma – coagulatory factors | 40355 |
| Factor VIII | 47032 |
| Factor VIII | 40303 |
| Factor VIII with dextrose | 40316 |
| Tranexamic (acide) | 41928 |
| Infections | |
| Diagnostic codes:  ICD-9 codes: 590.0-590.9, 599.0-599.6, 646.6, 595.0, 595.9, 646.5, 595.8, 460-466, 472-487  ICD-10 codes: O230-O239, N800-N809, N760-N763, N800-N809, N300, N309, N110, N111, N118, N119, N136, N158, N160, N161, N164, N165, N159, N390, N360- N363, N138, N139, A600, A609, A630, B333, B334, B338, B342-B344, B348, B971-B977, B349, N341, A540, A541, A549, A542, T520, A510, A519, A527, B079, A749, A748, A561, A590, N771, N16, N10, N12, O23, B20, J22, J31, J32, J39, J00-J06, J09-J18. |  |
| Medication generic codes:  Generic name | Quebec generic code |
| Cefprozil | 47139 |
| Ceftriaxone | 47669, 45548 |
| Ceftobiprole medocaril | 47761 |
| Cefsulodine (sodium) | 45113 |
| Ceftazidime (pentahydrate) | 45152 |
| Ceftriaxone (disodium) | 45473 |
| Cefixime | 45546 |
| Ceftazidime | 45615 |
| Cefprozil | 47139 |
| Ceftobiprole medocaril | 47761 |
| Cefsulodine (sodium) | 45113 |
| Ceftazidime | 45152 |
| Gentamycin | 46016, 46704 |
| Azitrhomycin | 46236,  47099 |
| Paroymycin | 46239, 46571,  47121 |
| Erythromycin | 46253, 46392  46393, 46611, 03458  03471, 03484, 03497, 03510, 03523, 38860, 44775 |
| Telithromycin | 47471 |
| Kanamycin | 05122 |
| Lincomycin | 05304 |
| Tobramycin | 47900, 33816, 42877 |
| Neomycin | 06409, 06422 |
| Spectinomycin | 09074 |
| Spiramycin | 09087 |
| Streptomycin | 09139 |
| Clarithromycin | 47018 |
| Clindamycin | 46600, 46807, 02041,  02054, 10686 |
| Fosfomycin Tromethamine | 46609, 47967 |
| Capréomycine (sulfate de) | 01378 |
| Dactinomycine | 02405 |
| Triamcinolone (acetonide)/ neomycin (sulfate)/ nystatin/gramicidin | 46274 |
| Vancomycin | 10036 |
| Bleomycin | 10569 |
| Mitomycin | 37690 |
| Chlortetracyclin | 01963 |
| Demethylchlortetracyclin | 02509 |
| Oxytetracyclin | 06890, 06903 |
| Tigecycline | 47656 |
| [Nitrofurantoin](https://www.drugs.com/nitrofurantoin.html) | 47081 |
| Amoxicillin | 18244 |
| Amoxicillin/ clavulanate potassium | 44333, 46515 |
| Ampicillin | 00533, 00546 |
| Carbenicillin | 01443 |
| Cloxacillin | 02106 |
| Dicloxacillin | 02743 |
| Methicillin | 05980 |
| Nafcillin | 06318 |
| Oxacillin | 06773 |
| Penicillin | 07007, 07020, 07033, 07046, 07059, 07072 |
| Cephalexin | 01612 |
| Ciprofloxacin | 45503, 45619, 45503 |
| Norfloxacin | 45443 |
| Grepafloxacin | 46509 |
| Trovafloxacin | 46528, 46650,  47311, 47319 |
| Moxifloxacin | 46698, 47391 |
| Ofloxacin | 45604 |
| Gatifloxacin | 46725, 47399 |
| Levofloxacin | 47258 |
| Gemifloxacin | 47721 |
| Norfloxacin | 45443 |
| Metronidazole | 46014, 46330, 47838,  06266 |
| Sulfamethoxazole trimethoprim | 41603 |
| Obesity | |
| Diagnostic codes:  ICD-9 codes: 278, 278.1, 278.8, 278.9, 649.1 ICD-10 codes: E660-E669 and R632 |  |
| Smoking | |
| Diagnostic codes:  ICD-9 codes: 649.0  ICD-10 codes: O993 | |
|  | |

# Table S5. List of diagnostic codes (ICD-9 and ICD-10) for pregnancy complication compared in Table 1.

Note. Pregnancy complications were compared between term and preterm birth groups but not used as covariates in multivariate analyses.

| Disease | ICD-9 codes | ICD-10 codes |
| --- | --- | --- |
| Premature rupture of membranes | 658.1 | O42.0-O42.9 |
| Placental dysfunction | 641.0-641.2, 656.7 | O44.0, O44.1, O45.0-O45.9 |
| Preterm labor | 644.0, 644.1, 644.9 | O60.0-O60.2 |
| Bleeding | 640.0-640.6,  641.8, 641.9 | O20.0-O20.9, O46.0-O46.9 |
